# The pathways from perceived discrimination to self-rated health among the Chinese diaspora during the COVID-19 pandemic: investigation of the roles of depression, anxiety, and social support

**DOI:** 10.1186/s12939-021-01537-9

**Published:** 2021-08-28

**Authors:** Youli Chen, Zicong Wang, Weizhen Dong, Jia Huei Chen Xu, Sizhe Ji Wu, Xiangyang Zhang, Chun Chen

**Affiliations:** 1 Wuhan Union Hospital, Tongji Medical College, Huazhong University of Science and Technology, Wuhan, 430000 China; 2 Department of Sociology and Legal Studies, University of Waterloo, 200 University Avenue West, Waterloo, ON N2L 3G1 Canada; 3 School of International Studies, Wenzhou Medical University, Wenzhou, 325035 China; 4 International School of Capital Medical University, Capital Medical University, Beijing, 100069 China; 5 Purchasing Department, First Affiliated Hospital of Wenzhou Medical University, Wenzhou, 325000 China; 6 School of Public Health and Management, Wenzhou Medical University, Wenzhou Medical University Chashan Campus, Tongren Building 7B304, Wenzhou, 325035 China

**Keywords:** Chinese diaspora, COVID-19, Self-rated health, Perceived discrimination, Mental health, Social support, Cross-sectional, Structural equation model

## Abstract

**Background:**

Research indicates the adverse impacts of perceived discrimination on health, and discrimination inflamed by the COVID-19 pandemic, a type of social exclusion, could affect the well-being of the Chinese diaspora. We analyzed the relationship and pathways of perceived discrimination’s effect on health among the Chinese diaspora in the context of the pandemic to contribute to the literature on discrimination in this population under the global public health crisis.

**Methods:**

We analyzed data from 705 individuals of Chinese descent residing in countries outside of China who participated in a cross-sectional online survey between April 22 and May 9, 2020. This study utilized a structural equation model (SEM) to evaluate both direct and indirect effects of perceived discrimination on self-rated health (SRH) and to assess the mediating roles of psychological distress (namely, anxiety and depression) and social support from family and friends.

**Results:**

This online sample comprised predominantly young adults and those of relatively high socioeconomic status. This study confirmed the total and direct effect of recently perceived discrimination on SRH and found the indirect effect was mainly mediated by depression. Mediating roles of anxiety and social support on the discrimination-health relationship were found insignificant in this SEM.

**Conclusions:**

Our findings suggest discrimination negatively affected the well-being of the Chinese diaspora, and depression acted as a major mediator between the discrimination-health relationship. Therefore, interventions for reducing discrimination to preserve the well-being of the Chinese diaspora are necessary. Prompt intervention to address depression may partially relieve the disease burden caused by the surge of discrimination.

**Supplementary Information:**

The online version contains supplementary material available at 10.1186/s12939-021-01537-9.

## Background

Discrimination is unfair treatment of a perceived group that often results from stigma, or prejudice, which is a negative or hostile attitude based mostly on false or incomplete information [1]. A major form of discrimination is based on race; it consists of beliefs, attitudes, and practices that harm individuals or groups because of their physiological features, place of origin, or culture and heritage [2].

Although the Chinese diaspora constitutes a significant part of the world’s immigrant population, it is a target for marginalization, stereotyping, and discrimination. In 2007–2008, it was clearly estimated for the first time that the number of Chinese diaspora was approximately 50 million [3], and the number raised to about 69 million in 2017. Apart from 70.4% of Chinese immigrants living in other Asian countries, the distribution of this population in 2018 was mainly in America (19.6%), Europe (4.6%), Oceania (3.2%), and Africa (2.3%) [4]. Asians, especially those of Chinese descent, face increased incidences of COVID-related racial discrimination, ranging from verbal and physical assaults to vandalism and workplace harassment, regardless of their actual disease status. The situation that Asians face today parallels past episodes of discrimination targeted at ethnic minorities related to infectious diseases [5]. In the COVID-19 era, various types of racism (e.g., explicit, implicit, institutional, symbolic) are integrative in understanding profound disparities [6], including those related to health [7].

The evidence of the link of racism on minority health outcomes is staggering. In a review of 138 empirical studies, Paradies [8] reported that 72% of the studies found a significant relationship between self-reported racism and mental health and 62% found physical health-related outcomes, including increased risk of cardiovascular, endocrine, and immune response diseases. Subjective perceptions of self-rated health (SRH) could be used for predicting the above-mentioned mental and physical health trajectories and is a powerful predictor for future mortality and morbidities [9].

This study attempts to understand whether subjective discriminatory experiences in the COVID-19 pandemic have negative implications for one’s health as measured by SRH. Although the existing literature has focused mainly on the chronic impact of long-term discrimination on health, we assumed that a burst of recent discrimination inflamed by the COVID-19 pandemic could lead to acute health deterioration. This assumption is based on a meta-analytic review done by Pascoe & Richman [10], which revealed that recent discrimination (discrimination one has experienced in the past year), compared with lifetime discrimination (discrimination one has experienced over one’s lifetime), had a more significant negative effect on overall well-being, including mental health and physical health.

Prior studies have found a significant cause-and-effect relationship between perceived racial discrimination and poorer SRH among various ethnic minorities, including Asians [11]. However, such research specific to the Chinese diaspora is lacking, especially in the context of COVID-19. Chinese have been subjected to rising overt discrimination and violent hate crimes, highlighting perception of discrimination, and in turn exacerbate the health implications of discrimination. Thus, we propose that perceived discrimination during the COVID-19 pandemic has a direct and negative impact on SRH (Hypothesis 1).

Although previous literature has left us with an in-depth investigation of the directions and pathways of discrimination-health relationships, these results rarely include Asian populations and could prove inconclusive in current situations. Existing literature has proposed behavioral patterns/responses (e.g., health practices, everyday resistance), psychological responses (e.g., internalized racism, racial identity, self-esteem, stereotype threat), physiological responses, and collective and individual resilience as mediators in the link between perceived discrimination and health [12]. In this study, we utilize SEM to understand the indirect effects of COVID-related discrimination on health within a biopsychosocial framework, which is widely used in explaining health disparities in minorities [13, 14]. Specifically, racial discrimination as a stressor elicits psychological stress responses and coping responses, such as mobilization of social resources, leading to health outcomes. We propose psychological distress (e.g., depression, anxiety) and social support as potential powerful mediators in the pathway from perceived discrimination to SRH, which have been subjected to major changes in the face of the COVID-related stigma, distancing regulations, the adaptive lifestyles, and the pandemic itself.

There is preliminary evidence that psychological mediators, including elevated stress and depressive symptoms [15], can indirectly affect the relationship between perceived discrimination and health conditions. Notably, the Chinese diaspora is suffering from prominent stigma-associated psychological stress during the COVID-19 pandemic [16], and the role of mental health status in the discrimination-health relationship should be investigated promptly. We hereby propose Hypothesis 2 as follows: beyond the direct and negative association with SRH, perceived discrimination influences SRH indirectly via psychological distress, namely, anxiety and depression. Specifically, anxiety and depression are consequences of discrimination and contribute to the negative assessment of SRH.

In a previous study by Yang & Chen [17], social capital, defined as the interactional and resourceful social relationships, buffered the adverse association of perceived discrimination with stress, one of the socio-attitudinal pathways linking perceived discrimination and health in ethnic minorities. There is a strong rationale for expecting that discrimination will lead to decreased social support, probably caused by a limitation of one’s social interactions to avoid repeatedly experiencing discrimination [18], and that decreased social support is closely associated with poorer general health and SRH [19]. During COVID-19, social support decrease could be exacerbated by requirements for ‘social isolation’ and hypervigilance in interpersonal contact for COVID self-protection. It is noteworthy that during the COVID-19 pandemic, social support from friends and family could be widely heterogenous, because domestic dynamics could rarely be affected by COVID-related racial discrimination, while people’s interaction with friends could be subject to changes. Thus, we hypothesize that after controlling for other covariates, the relationship between perceived discrimination and SRH is mediated by social support from family and friends. Explicitly, we expect the level of social support from friends to decrease when perceived discrimination increases, while that from family unaffected, and the lack of both sources of social support (family and friends) would lead to lower SRH (Hypothesis 3).

**Fig. 1:**
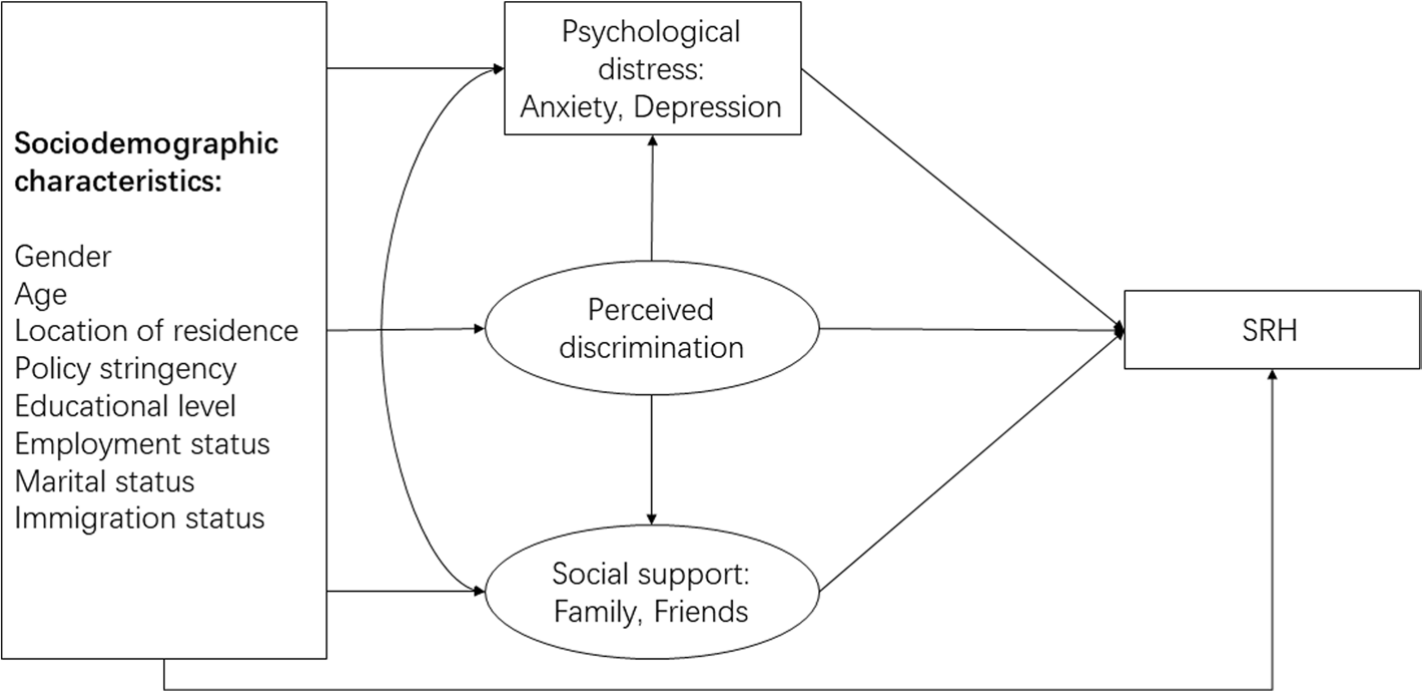
Conceptual framework and hypotheses. Notes: Rectangles indicate the manifest variables, and ovals indicate the latent variables

Moreover, we propose social support is correlated with psychological distress, in consistence with existing research [20, 21], which is reflected in our proposed model (Fig. 1).

Aside from the above major factors incorporated in this conceptual framework, socio-demographic characteristics have also been recognized as significant determinants of health. A large body of literature on various samples has found an association between SRH and socio-demographic characteristics. Female gender, older age, a lower level of education [22], lower income [23], not being married or living alone [24], were important factors associated with general health in European countries. Specific to immigrants, older age and a lower level of attained education was found to be associated with poor SRH in female Ukraine immigrants in Czechoslovakia [25]. Hong & Lee [26] found SRH was associated with the type of migration. Given the specific context of COVID-19, variations in the policy regarding containment and closure, including stay-at-home orders and policies limiting daily activities and gatherings, could also exert an influence on the accessibility of social support as well as mental health status. Thus, demographic and psychosocial factors were included in this model to control their potentially confounding effects on other concepts in the analytic framework.

Sparse literature has focused on the health consequences and mechanisms of spiking discrimination toward Chinese during the pandemic, although there have been calls for awareness of stigma and incorporation of coronavirus-related measures or methods into studies. This pandemic is a historical moment that will have a lasting effect on interpersonal and intercultural relations, which merits in-depth research [27]. COVID-related discriminatory events against the Chinese are rising, becoming more overt, and are “recent discrimination”, which is different from “lifetime discrimination” in nature. These features of COVID-related discrimination could impact SRH differently than that prior to the pandemic.

The current study was carried out in the first-wave, early, escalating stage of the COVID-19 pandemic. As the pandemic evolves, several countries are taking a hit of even second-wave pandemic already, which indicates the inevitability of the prolonged co-existence of humans and the coronavirus. Improved understanding of the mechanisms underlying the discrimination-health association would be valuable to inform intervening efforts to reduce stigma and disparities towards ethnic minorities [28], such as those of Chinese descent, and equipping the world to better adapt to public health challenges.

## Methods

### Data

Data in this study were collected by a multi-country, cross-sectional online survey of the Chinese diaspora residing in countries during the Covid-19 pandemic. Respondents were from all continents (except for Antarctica), which included ‘epicenter’ countries (e.g., the US, the UK, Spain, etc.) and others (e.g., Japan, Australia, etc.). The survey was carried out between April 22 and May 9, 2020.

The study sample was formed by a combination of snowball sampling and random sampling. Based on an online crowdsourcing platform powered by www.wenjuan.com, a part of the survey sample was collected from participants by targeted snowball sampling using a dominant social media platform in China, WeChat, with a link of the online questionnaire attached to each invitation. In addition, the survey collected a random sample using the corporate mailing list of dingwei.netease.com, a survey company, via e-mail with a link to the web-based survey. Among the mailing list, 3,194 of approximately 1,000,000 users who fitted the requirement for participation in the survey were randomly selected and sent e-mails. A total of 1,045 respondents completed the survey (328 from snowball sampling and 717 from random sampling). The snow-ball sampling precludes estimating a response rate, while the response rate of random sampling using emails was 22.4%, which is typical for online surveys [29]. Afterward, the invalid questionnaires were screened by C.Y. (missing values ≥ 15% of question items, answering time ≤ 3 s per question item), which resulted in a valid analytic sample comprised of 705 responses.

### Measures

#### SRH

The end outcome variable, SRH, was obtained by a single item asking participants to rate their general health status on a vertical, 0–100-point EuroQol visual analogue scale (EQ-VAS). The EQ-VAS rated the health status with endpoints of “best imaginable health state” set at 100 and “worst imaginable health state” set at 0. The EQ-VAS is a widely used instrument to describe individuals’ self-rated health and showed sufficient responsiveness [30], validity, and test–retest reliability [31] in the Chinese population.

#### Perceived discrimination

The latent perceived discrimination construct was comprised of five items adopted from the Everyday Discrimination Scale (EDS) (Short Version) [32] and modified to apply to the COVID-19 pandemic setting (Supplementary Table 1). The original study used this scale in a community sample of black, Hispanic, and white adults and achieved acceptable reliability (Cronbach’s α = 0.77). This scale was selected because it is a widely used, validated measure in identifying ethnically different causes and consequences of perceived discrimination in Asians [33, 34].

In our modified version, the construct describes five examples of discrimination (e.g., treated with less courtesy/respect than other people poorer service than others at restaurant/store) and asked respondents to indicate how often they encountered these situations in their everyday life since the outbreak of COVID-19 based on a 5-point Likert scale (0 = never, 4 = always). The five items of perceived discrimination had an internal reliability of 0.89, and removing any of them would reduce Cronbach’s Alpha Coefficient.

#### Anxiety and depression

Anxiety and depression were dichotomous variables. Specifically, the Generalized Anxiety Disorder Scale (GAD) -2 and the Patient Health Questionnaire (PHQ) -2 were used to assess the presence of anxiety and depressive symptoms experienced by participants over the past two weeks [35], separately. The GAD-2 included the items ‘feeling nervous, anxious, or on edge’ and ‘not being able to stop or control worrying’. The PHQ-2 included the items ‘little interest or pleasure in doing things’ and ‘feeling down, depressed, or hopeless’. Responses to each item were coded as 0 = not at all, 1 = several days, 2 = more than half the days, and 3 = nearly every day. Scores of each scale were summed with a potential range of 0 to 6, where a higher score indicated a higher degree of anxiety or depression. In this study, both the GAD-2 (Cronbach’s α = 0.83) and PHQ-2 (Cronbach’s α = 0.75) achieved acceptable reliability. We further dichotomized anxiety or depression as 1 when the scale score was ≥ 3, otherwise as 0, based on the suggested cut-off point by Kroenke et al. [35], where a scale score ≥ 3 suggested a likely condition of anxiety or depression. These scales were developed to screen for depression and anxiety disorders and were selected for their brevity and high sensitivity and specificity [36]. They were validated and standardized in the general population in Germany [36] and Columbia [37], and in healthcare workers in China [38], etc.

#### Social support

Social support is a latent construct measured by 6 modified items adopted from an abbreviated version of the Lubben Social Network Scale (LSNS-6) [39]. Both the original version and the modified version consisted of subscales considering social contact with family members and friends. The response categories were none, one, two, 3 or 4, 5–8, and 9 or more, which were assigned scores of 0–5, respectively. Compared with the original scale, both online or in-person social contact were included to fit the current pandemic and the widened age range of participants. Although this scale has been commonly used among community-dwelling elders because Lubben found that marital status and participation in religious activities vary less in the older adult population. Therefore, this measure focuses more heavily on the quality and frequency of an individual’s relationships with family and friends [39, 40]. This brief instrument fitted our purpose to specifically and separately gauge objective social isolation as well as perceived social support from family and friends, interactions with whom subject to drastic change during the pandemic. The composite reliability of the modified LSNS-6 was 0.828, and the reliability of the questions for the family and the friend subscales were 0.75 and 0.81, respectively.

#### Socio-demographic characteristics

A set of socio-demographic characteristics were included in the model as control variables in each structural equation.

Dichotomous variables include gender (1 = male, 0 = female), location of residence (1 = Asian countries, 0 = Non-Asian countries), employment status (1 = currently employed [employed full-time/part-time/self-employed], 0 = currently unemployed [student/retired/unable to work]), marital status (1 = married [common law or legally] or living with a partner, 0 = other statuses [e.g., single, divorced, or others]), and immigration status (1 = non-immigrant, 0 = immigrant [citizen/LPR [legal permanent resident]/CPR [conditional permanent resident]. Age was an ordinal variable. Educational level was categorized into three ordinal groups: ≤ 12 years, ≤ 15 years, and > 15 years.

An indicator for the stringency of government responses to COVID-19 was proposed by Oxford University, which quantifies variation in containment and closure policies, including stay-at-home orders and policies limiting daily activities and gatherings [41]. We adopted policy stringency in the proposed model as a continuous variable for its potential impact on health and social support parameters. Respondents were assigned a 0–100 stringency index number corresponding to their location on April 22^nd^, as calculated by the Oxford COVID-19 Government Response Tracker (OxCGRT).

### Data analysis

First, descriptive statistics were generated for all variables. Normality was tested using skewness and kurtosis of distribution [42]. In the analysis, either an absolute skew value larger than 2 or an absolute kurtosis larger than 7 were used as reference values for determining substantial deviation from normality. The original scores of all variables were all normally distributed.

Next, to explore the relationships among the study variables, we first entered all proposed measures into a correlation matrix to identify significant bivariate relationships. Two-tailed tests were utilized (*p* ≤ 0.05) to identify significant associations. If a variable was directly correlated with any of the variables on the left-hand side or variables were significantly associated with variables on the left-hand side, this information was used to develop an initial hypothesized model.

Finally, the main analysis was a multiple mediator analysis. The models were analyzed using Mplus Version 7 [43]. To test our framework in Fig. 1, a structural equation model (SEM) with latent variables was utilized to test the 3 hypotheses simultaneously. Each model was adjusted for gender, age, location of residence, policy stringency, educational level, employment status, marital status, and immigration status. Indirect effects were tested using a bootstrapping procedure using 5,000 resamples from the data set.

This study followed Muthén & Muthén [43] to report model-fit indices including Chi-square/df, Comparative Fit Index (CFI), the Tucker-Lewis Index (TLI), and the Root Mean Square Error of Approximation (RMSEA). Chi-square/df < 3 indicates an acceptable fit. RMSEA values of 0.08 or lower are indicative of a good fit. CFI values greater than roughly 0.90 may indicate a reasonably good fit of the model, though a value of 0.95 is preferable. TLI values of 0.90 or higher indicate incremental fit, and TLI values exceeding 0.95 indicate good model fit [44, 45]. We improved model fit during the estimation process by freeing covariances between error terms of the 5 measures of perceived discrimination and 6 measures of social support.

Six structural equations were specified with each variable on the left-hand side of the equation corresponding to one of the six key endogenous variables identified in the conceptual framework depicted in Fig. 1.This study followed a two-step estimation procedure as suggested by Anderson and Gerbing [46]. In the first step, confirmatory analysis and model fit statistics were used to establish an acceptable measurement. In the second step, the modified measurement model and the structural equations were estimated simultaneously. We employed the mean- and variance-adjusted weighted least squares (WLSMV) with listwise deletion of missing values as the estimation method in both steps. When categorical outcome variables or a mixture of binary, ordinal categorical, and continuous outcome measures are included in SEM, estimation methods designed for categorical variables are recommended for less bias and in terms of model-data fit [47]. Listwise deletion for missing values was adopted because missing values relating to sociodemographic variables are not random, and any filling-in method could cause false estimations.

This study intended to assess theoretical and context-based hypothesized associations and pathways between perceived discrimination and SRH and to explore the associations between the demographic variables and perceived discrimination, psychological distress, social support, and SRH. Thus, the study design consisted of both exploratory and confirmatory data analysis.

## Results

### Descriptive statistics

Descriptive data of all manifest variables in the analysis are summarized in Table 1. A total of 705 individuals completed the survey. Among respondents, 52.3% were males, 83.4% were currently living outside of Asia, 78.0% had received some post-secondary education (> 12 years), and 36.5% were married or living with a partner. 41.9% of participants were employed currently. As for immigration status, 46.1% were citizens, legal permanent residents, or conditional permanent residents. The majority of respondents were 18–40 years of age (83.1%).

**Table 1:** Descriptive statistics of the manifest variables obtained from the survey

	N, No. (%)	Mean, mean ± SD
SRH^a^ (0–100)	705 (100%)	85.97 ± 13.06
Perceived discrimination	705 (100%)	
PD1^b^ (0–4)	705 (100%)	2.14 ± 1.00
PD2^b^ (0–4)	705 (100%)	1.88 ± 0.98
PD3^b^ (0–4)	705 (100%)	2.02 ± 1.05
PD4^b^ (0–4)	705 (100%)	2.02 ± 1.03
PD5^b^ (0–4)	705 (100%)	1.73 ± 0.95
Anxiety (0 = NO, 1 = YES)	705 (100%)	0.3 ± 0.46
0 (REF)	495 (70.2%)	
1	210 (29.8%)	
Depression (0 = NO, 1 = YES)	705 (100%)	0.26 ± 0.44
0 (REF)	525 (74.5%)	
1	180 (25.5%)	
Social support	705 (100%)	
Social support from family	705 (100%)	
SS1^c^ (0–5)	705 (100%)	3.69 ± 1.35
SS2^c^ (0–5)	705 (100%)	3.13 ± 1.32
SS3^c^ (0–5)	705 (100%)	3.51 ± 1.31
Social support from friends	705 (100%)	
SS4^c^ (0–5)	705 (100%)	4.00 ± 1.36
SS5^c^ (0–5)	705 (100%)	3.48 ± 1.30
SS6^c^ (0–5)	705 (100%)	3.58 ± 1.38
Gender	701 (99.4%)	0.53 ± 0.50
Male	369 (52.3%)	
Female (REF)	332 (47.1%)	
Age	700 (99.3%)	2.88 ± 1.26
< 18	36 (5.1%)	
18–25	324 (46.0%)	
26–30	145 (20.6%)	
31–40	116 (16.5%)	
41–50	52 (7.4%)	
51–60	16 (2.3%)	
> 60	11 (1.6%)	
Location of residence		0.17 ± 0.37
Asian countries	117 (16.6%)	
Non-Asian countries (REF)	588 (83.4%)	
Policy stringency, mean (SD)	704 (99.9%)	76.13 ± 13.27
Educational level	689 (97.7%)	2.27 ± 0.78
≤ 12 years	139 (19.7%)	
≤ 15 years	227 (32.2%)	
> 15 years	323 (45.8%)	
Employment status	683 (95.5%)	0.51 ± 0.5
Currently employed (employed full-time/part-time/self-employed)	346 (49.1%)	
Currently unemployed (student/retired/unable to work) (REF)	337 (47.8%)	
Marital status	673 (95.5%)	0.38 ± 0.49
Married/ Living with a partner/ Common law	257 (36.5%)	
Unmarried (Single/Other) (REF)	416 (59.0%)	
Immigration status	668 (94.8%)	0.51 ± 0.50
Immigrant (Citizen/LPR^d^/CPR^e^) (REF)	325 (46.1%)	
Non-immigrant	343 (48.7%)	

^a^*SRH* self-rated health, ^b^*PD* Perceived discrimination, ^c^*SS* Social support, ^d^*LPR* Legal permanent resident, ^e^*CPR* Conditional permanent resident

Participants’ mean score of SRH is 85.97 ± 13.06. Anxiety (29.8%) and depressive symptoms (25.5%) were relatively prevalent among participants.

### Correlation matrix

The significant associations identified in Supplementary Table 3 indicate that SRH was negatively associated with all 5 manifest variables in the perceived discrimination construct, anxiety, and depression, and positively associated with frequent contact with family members and the number of trusted relatives. Also, the 5 manifest variables in the perceived discrimination construct were positively associated with the likelihood of anxiety and depression. Manifest variables in the perceived discrimination construct and manifest variables in the social support construct of statistical significance all indicate converse relationships.

Outside the variables in our initial hypotheses, we also found SRH negatively associated with educational level. Older people, those currently employed, and married people were more likely to have anxiety.

### SEM Results

In the analysis of the path model (Fig. 1), goodness-of-fit indices for the model indicated a good fit. Specifically, the Chi-square/df value (243.218/129 = 1.885) for the model was lower than 3, the CFI value (0.941) and the TLI value (0.907) for the model were higher than 0.90, and the RMSEA value (0.037) was lower than 0.05, suggesting a good fit of the model.

The associated confirmatory factor analysis results were presented in Supplementary Table 4. All standardized factor loadings of manifest variables in latent constructs were nontrivial and significant (*p* < 0.001). Several paths in the model were not statistically significant: from perceived discrimination to social support from family (*p* = 0.057), from social support from friends to SRH (*p* = 0.089), and from anxiety to SRH (*p* = 0.807). However, all paths were retained in the final model, given the model integrity and adequate model fit.

**Fig. 2:**
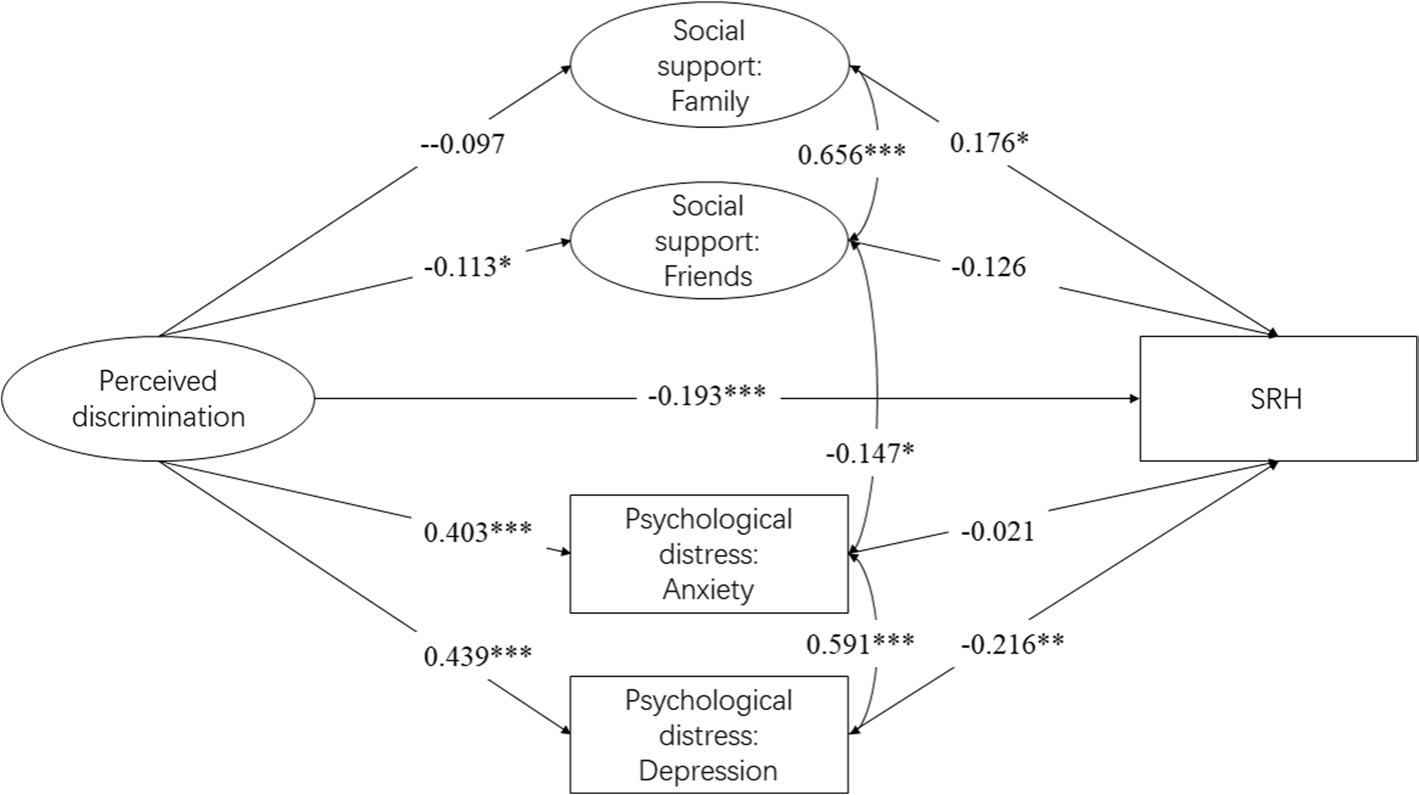
Observed SEM of perceived discrimination on SRH. Notes: numbers in the figure along any single-headed arrow are the standardized path coefficients. *, *p* < 0.05; **, *p* < 0.01; ***, *p* < 0.001

Table 2 illustrates standardized path coefficients corresponding to Fig. 2, the variables on the left-hand side being endogenous variables: perceived discrimination, depression, anxiety, social support from family and friends, and SRH. Substantive findings are summarized as follows.

**Table 2:** Standardized path coefficients from the SEM

Variables on the left-hand side →	**(1)**	**(2)**	**(3)**	**(4)**	**(5)**	**SRH**^a^
Variables on the right-hand side ↓						
**Perceived discrimination**		0.439***	0.403***	-0.097	-0.113*	-0.193***
**Depression**						-0.216**
**Anxiety**						-0.021
**Social support from family**						0.176*
**Social support from friends**						-0.126
**Sociodemographic characteristics**						
Male	0.106*	-0.076	-0.028	-0.074	-0.115*	0.024
Age	-0.237***	-0.092	0.098	0.173*	0.101	-0.058
Location of residence	0.001	0.092	-0.008	-0.066	-0.102*	0.088*
Policy stringency	-0.061	0.078	0.063	0.058	0.051	0.044
Educational level	-0.012	-0.015	-0.077	0.042	0.162**	-0.017
Employed	0.125*	< 0.0001	0.059	0.050	0.041	0.023
Married	0.150*	0.082	0.059	0.129	-0.034	-0.081
Non-immigrant	0.035	0.021	0.155*	0.099	0.066	-0.076

^*^, *p* < 0.05; **, *p* < 0.01; ***, *p* < 0.001

^a^*SRH* Self-rated health

First, we found modulating effects of several sociodemographic factors on endogenous variables. Males, younger people, the employed, and married people reported more encounters of discrimination.

No sociodemographic characteristics were found correlated to depression, while non-immigrants were more likely to exhibit anxiety symptoms. After accounting for sociodemographic characteristics, perceived discrimination significantly led to depression and anxiety, lending support to Hypothesis 2.

The fourth and fifth columns demonstrate the results using social support as the dependent variable. A higher level of social support from family was anticipated by older age, while females, people living outside of Asia, and people who attained a higher level of education possessed more friendship-related social support. Perceived discrimination was found to be, as expected, negatively associated with social support from friends, which partially supported our Hypothesis 3.

Finally, SRH was the dependent variable in the sixth column that allowed us to examine the remaining hypotheses. First, after accounting for other covariates, perceived discrimination was found negatively associated with SRH, which is in accordance with Hypothesis 1. Second, the negative relationship between depression and SRH partially confirmed our Hypothesis 2 but the association between anxiety and SRH was not significant in this SEM. Third, a higher level of social support from family leads to better SRH. In contrast, the relationship between the level of social support from friends and SRH is statistically insignificant, partly contradicting our Hypothesis 3. Also, living in Asian countries was found to be positively associated with SRH.

The direct and indirect effects of perceived discrimination on SRH are summarized in Table 3. Overall, both direct and indirect effects of perceived discrimination on SRH were significant. The magnitude of direct effect (-0.193) was higher than indirect effect (-0.106), indicating perceived discrimination’s strong and direct impact on SRH. The indirect effect was solely significantly contributed to depression (-0.095). When indirect effects were tested by bootstrapping method, family-related support presented with potential negative mediating effects (*p* = 0.153, 95%CI: -0.045, -0.004), while friendship-related support presented with potential positive mediating effects (*p* = 0.189, 95%CI: 0.001, 0.037), which warrants future investigation. While perceived discrimination negatively contributed to anxiety, the effects of anxiety on SRH were not significant, which could account for the finding that indirect effects through anxiety were not significant.

**Table 3:** Summary of standardized direct and indirect effects of discrimination on SRH

	**Coefficient**	***p*-values**	**95%CI**
Total effect	-0.299	< 0.001	(-0.377, -0.214)
Direct effect	-0.193	< 0.001	(-0.274, -0.108)
Total indirect effect	-0.106	< 0.001	(-0.156, -0.062)
Indirect effect via			
Depression	-0.095	0.012	(-0.160, -0.036)
Anxiety	-0.009	0.809	(-0.069, 0.048)
Social support from family	-0.017	0.153	(-0.045, -0.004)
Social support from friends	0.014	0.189	(0.001, 0.037)

## Discussion

This study was the first to attempt to attain the SRH of the Chinese diaspora during the COVID-19 pandemic, to examine the associations of perceived discrimination and SRH in this sample and to assess whether this relationship was mediated by psychological distress and social support. Results yielded by the SEM indicate that perceived discrimination was both directly and indirectly associated with SRH, and that depression was a major mediator of this relationship.

This study adds to a growing literature in this area and extends findings supporting a link between recent discriminatory experiences and health deterioration among the Chinese diaspora, specific to the COVID-19 context. The size of the total and direct effects we observed is comparable to or larger than the size of effects reported in studies done in Asians prior to the pandemic in which we could make direct comparisons between standardized estimates or correlation coefficients [11, 48]. This is an important extension, as Asian and Chinese populations have been faced with a surge of discrimination since the COVID-19 outbreak, and this could pose challenges in areas relating to public health in the future. Moreover, the existence of significant relationships was established despite the particular characteristics of the current sample, which included participants who were relatively well-educated [49] and possibly of relatively high socioeconomic status. Notably, socio-demographic variables rarely affected SRH, in contrast with reports in studies before the pandemic. These results reflected on the ubiquity of racial discrimination and its impact on health, especially in the COVID-19 context.

Another potential contribution of this study lies in its findings of indirect effects. In this biopsychosocial model, the indirect effect of discrimination on health was found to be mediated by depression, which accounted for approximately 1/3 of the estimated variance of the association. Depression as a proximate mechanism through which discrimination affects SRH echoed the conclusion of a study conducted by Cuevas et al. [15] on African Americans, but with a larger indirect effect size. It is possible that discrimination-associated development, sustenance, and recurrence of depression [50] prominent in this pandemic sensitizes [51] and prolongs [52] the physiological responses to sources of stress sources. However, while there was a significant link between perceived discrimination and anxiety, similar to the findings of existing studies [53], anxiety was not a significant mediator between the discrimination-health relationship in this SEM, unlike in a previous study on African-American women [54]. A possible explanation could be the low degree of somatization of mental disorders among the Chinese. Assari [55] found ethnic variations in the correlations between mental health issues and SRH, where no significant correlations were found between anxiety and SRH among Chinese immigrants. Although emphasis has been put on investigating and preserving mental health of minority groups subject to discrimination in current context of the pandemic, this study adds yet another piece of evidence for mental health’s importance in not only itself, but also its effect on general health as a linkage in the discrimination-health relationship.

Furthermore, social support measured by the number of family members or friends participants frequently interacted with did not display a significant mediating effect on the relationship between perceived discrimination and health in the COVID-19 context possibly due to the complex and conflicting effect of social support on health. When deconstructed, discrimination did not affect social support from family members, which was obviously logical. Nevertheless, the potential health-promoting effect of social support from family members should be noted and utilized. However, while social support from friends correlated negatively with perceived discrimination, indicating a certain level of social withdrawal because of perceived discrimination among participants, it was associated negatively with SRH, contradicting the hypothesized buffering effect of social support from friends. This could be due to hostile or cynical responses provided by friends when participants were seeking social support, which may diminish the health benefits of social support [56]. Another explanation lies in the irrationality of contending that social interaction and social support are inherently good [57]. Increased conflict or shared health-compromising behaviors (e.g., tobacco and alcohol use) in social networks may result in adverse health outcomes [58].

However, several limitations of this study should be noted and the results be interpreted with caution. First, though the SEM in this study is recursive in nature, the present study was cross-sectional, and definitive causal relationships await longitudinal study designs. Second, the online survey sample was not entirely randomized and the responsive rate was relatively low, which is typical for online surveys, yielding results subject to coverage and volunteer bias. For example, young adults [18–25 years of age] were likely over-represented, comprising nearly half of the sample. However, despite these limitations, we managed to produce an extrapolatable sample of the Chinese diaspora with international coverage in a timely fashion that also adequately met the sample size requirements for SEM [59], although the Chinese diaspora is among the populations that are difficult to sample [60]. Last, the hypothesized mediators in this study were not exhaustive, although it fits roughly into the biopsychosocial framework and the measures used in this study are limited. The EQ-VAS for measurement of SRH, for example, is simple and easy for administration and scoring [61], its interpretation could vary across respondents [62], especially when interpreting extreme health states, which could lead to reference bias [63]. Future research could consider including other potentially influential covariates, moderator, and mediator variables, or adjusting the measurements used in this study.

## Conclusions

Although more research is needed to determine the causal relationship and pathways between perceived discrimination and health, prompt attention and intervention to address depression among the Chinese diaspora during the COVID-19 pandemic may partially relieve the disease burden caused by the surge of discrimination. Notably, this study provides new perspectives on the different roles of anxiety and depression and the varied roles of social support from different populations in mediation of the discrimination-health relationship specific to the COVID-19 context, indicating the uniqueness of this pandemic with regard to psychology, sociology, and health characteristics.

## Supplementary Information

**Additional file 1: Supplementary Table 1**. Questionnaire for investigation of the association between perceived discrimination and mental health status among the Chinese diaspora during the COVID-19 pandemic.

**Additional file 2: Supplementary Table 2**. Distribution of the manifest variables

**Additional file 3: Supplementary Table 3**. Correlation values for study variables

**Additional file 4: Supplementary Table 4**. Internal consistency and confirmatory factor analysis (CFA)

## Data Availability

The datasets used and analysed during the current study are available from the corresponding author on reasonable request.
