# Supplementary material for: The pathways from perceived discrimination to self-rated health among the Chinese diaspora during the COVID-19 pandemic: investigation of the roles of depression, anxiety, and social support
Source: Int J Equity Health. 2021 Aug 28;20:192. doi: 10.1186/s12939-021-01537-9 (PMC8401352; doi:10.1186/s12939-021-01537-9)
Supplement: Supplementary file 1 — Additional file 1: Supplementary Table 1. Questionnaire for investigation of the association between perceived discrimination and mental health status among the Chinese diaspora during the COVID-19 pandemic. [file 12939_2021_1537_MOESM1_ESM.docx]

Supplementary Table 1. Questionnaire for investigation of the association between perceived discrimination and mental health status among the Chinese diaspora during the COVID-19 pandemic

| Items | | Answer options |
| --- | --- | --- |
| Q1 | Gender: | O Male  O Female  O Other  O Prefer not to answer |
| Q2 | Age: | O 18 and younger  O 18-25  O 26-30  O 31-40  O 41-50  O 51-60  O 60 and older  O Prefer not to answer |
| Q3 | Location: (FILL IN THE BLANKS) | ________(Country)________(State/Province) |
| Q4 | Education: | O Less than high school  O High school, no diploma  O High school diploma  O Some college, no diploma  O Some university, no diploma  O College degree  O Bachelor degree and higher |
| Q5 | Employment status: | O Employed full-time  O Employed part-time  O Self-employed  O Unemployed  O Student  O Retired  O Unable to work  O Prefer not to answer |
| Q6 | Marital status: | O Married/ Living with a partner/ Common law  O Single (Never married /Widowed /Divorced /Separated)  O Other  O Prefer not to answer |
| Q7 | Immigration status: | O Citizen  O Legal Permanent Resident (e.g. “green card holder”, “blue card holder”)  O Conditional Permanent Resident  O Non-immigrant (e.g. visitors for business and for pleasure, students, temporary workers and trainees, treaty traders and investors, exchange visitors, religious workers, etc.)  O Other (asylee or refugee, undocumented person, person with temporary protected status, etc.)  O Prefer not to answer |
| Q8 | In your day-to-day life from 2019, Dec. up till now, how often have any of the following things happened to you? | O Never  O Seldom  O Sometimes  O Often  O Always |
| Q8.1 | You are not being treated with courtesy or respect as much as others. |  |
| Q8.2 | You have received poorer service than others restaurants or stores. |  |
| Q8.3 | People around you acted as if they wanted to avoid you. |  |
| Q8.4 | You have been called names or insulted by others. |  |
| Q8.5 | You are threatened or harassed. |  |
| Q9 | Please choose one point in 0–100, how would you assess your general health status now? |  |
| Q10 | Over the last 2 weeks, how often have you been bothered by the following problems? | O Not at all  O Several days  O More than half the days  O Nearly every day |
| Q10.1 | Feeling nervous, anxious, or on edge |  |
| Q10.2 | Not being able to stop or control worrying |  |
| Q10.3 | Little interest or pleasure in doing things |  |
| Q10.4 | Feeling down, depressed, or hopeless |  |
| Q11 | During the COVID-19 pandemic, | O None  O One  O Two  O 3 or 4  O 5-8  O 9 or more |
| Q11.1 | How many relatives do you see or hear from (online or in person) at least once a month? |  |
| Q11.2 | How many relatives do you feel at ease with that you can talk about private matters? |  |
| Q11.3 | How many relatives do you feel close enough that you could call on them for help? |  |
| Q11.4 | How many friends do you see or hear from (online or in person) at least once a month? |  |
| Q11.5 | How many friends do you feel at ease with that you can talk about private matters? | O None  O One  O Two  O 3 or 4  O 5-8  O 9 or more |
| Q11.6 | How many friends do you feel close enough that you could call on them for help? |  |
