# Supplementary material for: The pathways from perceived discrimination to self-rated health among the Chinese diaspora during the COVID-19 pandemic: investigation of the roles of depression, anxiety, and social support
Source: Int J Equity Health. 2021 Aug 28;20:192. doi: 10.1186/s12939-021-01537-9 (PMC8401352; doi:10.1186/s12939-021-01537-9)
Supplement: Supplementary file 2 — Additional file 2: Supplementary Table 2. Distribution of the manifest variables [file 12939_2021_1537_MOESM2_ESM.docx]

**Supplementary Table 2. Distribution of the manifest variables**

| **Manifest variables** | **Skewness** | **Kurtosis** |
| --- | --- | --- |
| **SRH**^a^ (0-100) | -1.559 | 5.168 |
| **PD1**^b^ (0-4) | 0.584 | -0.303 |
| **PD2** ^b^ (0-4) | 0.996 | 0.351 |
| **PD3** ^b^ (0-4) | 0.803 | -0.114 |
| **PD4** ^b^ (0-4) | 0.785 | -0.16 |
| **PD5** ^b^ (0-4) | 1.341 | 1.362 |
| **Anxiety** (0 = No, 1 = Yes) | 0.886 | -1.219 |
| **Depression** (0 = No, 1 = Yes) | 1.125 | -0.737 |
| **SS1**^c^ (0-5) | -0.375 | -0.332 |
| **SS2** ^c^ (0-5) | 0.037 | -0.615 |
| **SS3** ^c^ (0-5) | -0.094 | -0.404 |
| **SS4** ^c^ (0-5) | -0.407 | -0.339 |
| **SS5** ^c^ (0-5) | -0.024 | -0.448 |
| **SS6** ^c^ (0-5) | -0.087 | -0.602 |
| **Gender** (0 = Female, 1 = Male) | -0.106 | -1.994 |
| **Age** (1 = < 18 yo, 2 = 18-25 yo, 3 = 26-30 yo, 4 = 31-40 yo, 5 = 41-50 yo, 6 = 51-60 yo, 7= > 60 yo) | 1.054 | 0.798 |
| **Location of residence** (0 = Non-Asian countries, 1 = Asian countries) | 1.800 | 1.242 |
| **Policy stringency, mean (SD)** (0-100) | -0.506 | -0.591 |
| **Educational level** (1 = ≤12 years, 2 = ≤15 years, 3 = ＞15 years) | -0.501 | -1.17 |
| **Employment status** (0 = Currently unemployed [student/retired/unable to work]; 1 = Currently employed [employed full-time/part-time/self-employed]) | -0.026 | -2.005 |
| **Marital status** (0 = Unmarried [Single/Other]; 1 = Married/ Living with a partner/ Common law) | 0.487 | -1.768 |
| **Immigration status** (0 = Immigrant (Citizen/LPR^d^/CPR^e^); 1 = Non-immigrant) | -0.054 | -2.003 |

(a) SRH: self-rated health; (b) PD: perceived discrimination; (c) SS: social support; (d) LPR: legal permanent resident; (e) CPR: conditional permanent resident
