# Supplementary material for: The pathways from perceived discrimination to self-rated health among the Chinese diaspora during the COVID-19 pandemic: investigation of the roles of depression, anxiety, and social support
Source: Int J Equity Health. 2021 Aug 28;20:192. doi: 10.1186/s12939-021-01537-9 (PMC8401352; doi:10.1186/s12939-021-01537-9)
Supplement: Supplementary file 3 — Additional file 3: Supplementary Table 3. Correlation values for study variables [file 12939_2021_1537_MOESM3_ESM.docx]

**Supplementary Table 3.** **Correlation values for study variables**

|  | (1) | (2) | (3) | (4) | (5) | (6) | (7) | (8) | (9) | (10) | (11) | (12) | (13) | (14) | (15) | (16) | (17) | (18) | (19) | (20) | (21) | (22) |
| --- | --- | --- | --- | --- | --- | --- | --- | --- | --- | --- | --- | --- | --- | --- | --- | --- | --- | --- | --- | --- | --- | --- |
| 1. SRH^a^ | 1 | -0.240*** | -0.234*** | -0.277*** | -0.174*** | -0.258*** | -0.191*** | -0.231** | 0.036 | 0.075* | 0.099** | 0.004 | 0.023 | 0.008 | -0.002 | -0.043 | 0.026 | 0.059 | -0.102** | -0.011 | -0.068 | -0.061 |
| 1. PD1^b^ |  | 1 | 0.711*** | 0.653*** | 0.577*** | 0.577*** | 0.168*** | 0.214*** | -0.056 | -0.049 | -0.086* | -0.112** | -0.075* | -0.068 | 0.048 | -0.083* | 0.02 | -0.015 | -0.049 | 0.030 | -0.03 | -0.020 |
| 1. PD2 ^b^ |  |  | 1 | 0.664*** | 0.523*** | 0.607*** | 0.180*** | 0.229*** | -0.046 | 0.006 | -0.076* | -0.086* | -0.056 | -0.032 | 0.066 | -0.059 | 0.079* | -0.118** | 0.030 | 0.028 | 0.010 | 0.051 |
| 1. PD3 ^b^ |  |  |  | 1 | 0.576*** | 0.563*** | 0.200*** | 0.230*** | -0.109** | -.083* | -0.116** | -0.080* | -0.099** | -0.086* | 0.055 | -0.053 | 0.031 | -0.064 | 0.036 | 0.025 | 0.010 | 0.046 |
| 1. PD4 ^b^ |  |  |  |  | 1 | 0.658*** | 0.278*** | 0.293*** | -0.049 | -0.053 | -0.097* | -0.106** | -0.085* | -0.103** | 0.073 | -0.103** | -0.017 | 0.003 | -0.063 | 0.045 | -0.022 | -0.076 |
| 1. PD5 ^b^ |  |  |  |  |  | 1 | 0.243*** | 0.230*** | -0.098** | -0.037 | -0.133** | -0.126** | -0.104** | -0.099** | 0.127** | -0.065 | 0.052 | -0.138*** | 0.010 | 0.066 | 0.030 | 0.046 |
| 1. Anxiety |  |  |  |  |  |  | 1 | 0.422*** | 0.038 | 0.054 | -0.061 | -0.05 | -0.099** | -0.153*** | 0.009 | 0.103** | 0.1 | -0.003 | -0.026 | 0.087* | 0.095* | 0.021 |
| 1. Depression |  |  |  |  |  |  |  | 1 | -0.001 | -0.02 | -0.100** | -0.017 | -0.042 | -0.078* | -0.018 | -0.029 | 0.71 | -0.024 | -0.021 | 0.016 | 0.026 | 0.022 |
| 1. SS1^c^ |  |  |  |  |  |  |  |  | 1 | 0.541*** | 0.449*** | 0.411*** | 0.291*** | 0.223*** | -0.088* | 0.186*** | -0.067 | 0.074 | 0.055 | 0.101** | 0.152*** | -0.044 |
| 1. SS2 ^c^ |  |  |  |  |  |  |  |  |  | 1 | 0.513*** | 0.307*** | 0.432*** | 0.293*** | -0.009 | 0.247*** | -0.019 | -0.006 | 0.068 | 0.171*** | 0.230*** | 0.007 |
| 1. SS3 ^c^ |  |  |  |  |  |  |  |  |  |  | 1 | 0.348*** | 0.431*** | 0.475*** | -0.049 | 0.093* | -0.116** | 0.103** | 0.039 | 0.042 | 0.089* | -0.02 |
| 1. SS4 ^c^ |  |  |  |  |  |  |  |  |  |  |  | 1 | 0.522*** | 0.475*** | -0.127** | 0.028 | -0.105** | 0.096* | 0.052 | -0.019 | -0.013 | 0.009 |
| 1. SS5 ^c^ |  |  |  |  |  |  |  |  |  |  |  |  | 1 | 0.693*** | -0.093* | 0.106** | -0.098** | 0.022 | 0.162*** | 0.034 | 0.036 | 0.016 |
| 1. SS6 ^c^ |  |  |  |  |  |  |  |  |  |  |  |  |  | 1 | -0.053 | 0.052 | -0.089* | 0.003 | 0.155*** | -0.005 | 0.043 | 0.057 |
| 1. Gender |  |  |  |  |  |  |  |  |  |  |  |  |  |  | 1 | 0.058 | 0.07 | -0.119** | -0.010 | 0.144*** | 0.033 | -0.120 |
| 1. Age |  |  |  |  |  |  |  |  |  |  |  |  |  |  |  | 1 | -0.025 | -0.017 | 0.214*** | 0.553*** | 0.695*** | -0.281*** |
| 1. Location of residence |  |  |  |  |  |  |  |  |  |  |  |  |  |  |  |  | 1 | -0.491 | 0.099 | 0.081* | 0.008 | 0.175*** |
| 1. Policy stringency |  |  |  |  |  |  |  |  |  |  |  |  |  |  |  |  |  | 1 | -0.357*** | -0.023 | -0.026 | -0.341*** |
| 1. Educational level |  |  |  |  |  |  |  |  |  |  |  |  |  |  |  |  |  |  | 1 | 0.114** | 0.115** | 0.262*** |
| 1. Employment status |  |  |  |  |  |  |  |  |  |  |  |  |  |  |  |  |  |  |  | 1 | 0.543*** | -0.382*** |
| 1. Marital status |  |  |  |  |  |  |  |  |  |  |  |  |  |  |  |  |  |  |  |  | 1 | -0.304*** |
| 1. Immigration status |  |  |  |  |  |  |  |  |  |  |  |  |  |  |  |  |  |  |  |  |  | 1 |

*, p<0.05; **, p<0.01; ***, p<0.001

(a) SRH: self-rated health; (b) PD: perceived discrimination; (c) SS: social support
