# Supplementary material for: The pathways from perceived discrimination to self-rated health among the Chinese diaspora during the COVID-19 pandemic: investigation of the roles of depression, anxiety, and social support
Source: Int J Equity Health. 2021 Aug 28;20:192. doi: 10.1186/s12939-021-01537-9 (PMC8401352; doi:10.1186/s12939-021-01537-9)
Supplement: Supplementary file 4 — Additional file 4: Supplementary Table 4. Internal consistency and confirmatory factor analysis (CFA) [file 12939_2021_1537_MOESM4_ESM.docx]

**Supplementary Table 4. Internal consistency and confirmatory factor analysis (CFA)**

| **Latent construct** | **Manifest variable** | **Question in the survey** | **Internal consistency** | | **CFA** | |
| --- | --- | --- | --- | --- | --- | --- |
|  |  |  | **Cronbach’s Alpha** | **Alpha if item deleted** | **Factor loading** | **p-value** |
| **Perceived discrimination** |  |  | 0.891 |  |  |  |
|  | PD1^a^ | You are not being treated with courtesy or respect as much as others. |  | 0.865 | 0.786 | <0.001 |
|  | PD2 ^a^ | You have received poorer service than others restaurants or stores. |  | 0.858 | 0.806 | <0.001 |
|  | PD3 ^a^ | People around you acted as if they wanted to avoid you. |  | 0.864 | 0.802 | <0.001 |
|  | PD4 ^a^ | You have been called names or insulted by others. |  | 0.877 | 0.764 | <0.001 |
|  | PD5 ^a^ | You are threatened or harassed. |  | 0.869 | 0.800 | <0.001 |
| **Social support** |  |  | 0.828 |  |  |  |
| **Family subscale** |  |  | 0.754 |  |  |  |
|  | SS1 ^b^ | How many relatives do you see or hear from (online or in person) at least once a month? |  | 0.689 | 0.614 | <0.001 |
|  | SS2 ^b^ | How many relatives do you feel at ease with that you can talk about private matters? |  | 0.623 | 0.704 | <0.001 |
|  | SS3 ^b^ | How many relatives do you feel close enough that you could call on them for help? |  | 0.701 | 0.778 | <0.001 |
| **Friend subscale** |  |  | 0.807 |  |  |  |
|  | SS4 ^b^ | How many friends do you see or hear from (online or in person) at least once a month? |  | 0.567 | 0.683 | <0.001 |
|  | SS5 ^b^ | How many friends do you feel at ease with that you can talk about private matters? |  | 0.725 | 0.818 | <0.001 |
|  | SS6 ^b^ | How many friends do you feel close enough that you could call on them for help? |  | 0.681 | 0.754 | <0.001 |

(a) PD: perceived discrimination; (b) SS: social support
